# Assessment of *Helicobacter pylori* eradication in patients on NSAID treatment

**DOI:** 10.1186/1471-230X-12-133

**Published:** 2012-09-24

**Authors:** Harald E Vonkeman, HTJI deLeest, MAFJ van deLaar, J vanBaarlen, KSS Steen, WF Lems, JWJ Bijlsma, EJ Kuipers, HHML Houben, M Janssen, BAC Dijkmans

**Affiliations:** 1 Arthritis Center Twente, Department of Rheumatology and Clinical Immunology, Medisch Spectrum Twente Hospital and University of Twente, P.O. Box 50.000, 7500 KA, Enschede, The Netherlands; 2 Department of Rheumatology, VU University Medical Center and Jan van Breemen Institute, Amsterdam, The Netherlands; 3 Laboratorium Pathologie Oost-Nederland, , Enschede, The Netherlands; 4 Department of Rheumatology and Clinical Immunology, University Medical Center Utrecht, Utrecht, The Netherlands; 5 Department of Gastroenterology and Hepatology, Erasmus MC University Medical Center, Rotterdam, The Netherlands; 6 Department of Rheumatology, Atrium Medical Center, Heerlen, The Netherlands; 7 Department of Rheumatology, Rijnstate Hospital, Arnhem, The Netherlands

## Abstract

**Background:**

In this post-hoc analysis of a randomized, double blind, placebo controlled trial, we measured the sensitivity and specificity of *Helicobacter pylori* IgG-antibody titer changes, hematoxylin and eosin (H&E) stains, immunohistochemical (IHC) stains and culture results in NSAID using patients, following *H. pylori* eradication therapy or placebo.

**Methods:**

347 NSAID using patients who were *H. pylori* positive on serological testing for *H. pylori* IgG-antibodies were randomized for *H. pylori* eradication therapy or placebo. Three months after randomization, gastric mucosal biopsies were taken for *H. pylori* culture and histological examination. At 3 and 12 months, blood samples were taken for repeated serological testing. The gold standard for *H. pylori* infection was based on a positive culture or both a positive histological examination and a positive serological test. Sensitivity, specificity and receiver operating curves (ROC) were calculated.

**Results:**

*H. pylori* eradication therapy was successful in 91% of patients. Culture provided an overall sensitivity of 82%, and 73% after eradication, with a specificity of 100%. Histological examination with either H&E or IHC stains provided sensitivities and specificities between 93% and 100%. Adding IHC to H&E stains did not improve these results. The ROC curve for percent change in *H. pylori* IgG-antibody titers had good diagnostic power in identifying *H. pylori* negative patients, with an area under the ROC curve of 0.70 (95 % CI 0.59 to 0.79, *P* = 0.085) at 3 months and 0.83 (95% CI 0.76 to 0.89, *P* < 0.0001) at 12 months. A cut-off point of at least 21% decrease in *H. pylori* IgG-antibody titers at 3 months and 58% at 12 months provided a sensitivity of 64% and 87% and a specificity of 81% and 74% respectively, for successful eradication of *H. pylori*.

**Conclusions:**

In NSAID using patients, following *H. pylori* eradication therapy or placebo, histological examination of gastric mucosal tissue biopsies provided good sensitivity and specificity ratios for evaluating success of *H. pylori* eradication therapy. A percentual *H. pylori* IgG-antibody titer change has better sensitivity and specificity than an absolute titer change or a predefined *H. pylori* IgG-antibody titer cut-off point for evaluating success of *H. pylori* eradication therapy.

## Background

*Helicobacter pylori* (*H. pylori*) infection has been shown to be related to the development of peptic ulcer disease, chronic gastritis, MALT lymphoma and gastric cancer [1-4]. Accurate diagnosis of *H. pylori* infection has clinical consequences as *H. pylori* eradication improves outcome and recurrence of peptic ulcer disease. *H. pylori* infection can be detected using non-invasive tests such as serological tests, 13C-urea breath test and stool tests, and invasive tests requiring endoscopically obtained gastric mucosal tissue biopsies, such as tissue culture, examination of histological stains and the rapid urease test. Serological tests based on the detection of antibodies to *H. pylori* have been shown to have high sensitivity and are therefore useful in screening for *H. pylori* infection [5-7]. However, because serological tests merely detect an immune response, they do not discriminate between current or previous infection. *H. pylori* infection of the gastric mucosa causes a chronic local inflammatory cell infiltration, which in turn gives rise to a serological response, in which *H. pylori* specific antibodies are almost always detectable [8,9]. After successful *H. pylori* eradication therapy, the level of *H. pylori* specific antibodies decreases progressively over a period of several months, possibly parallel to the slowly healing inflammation of the gastric mucosa [10]. As a result, evaluating success of *H. pylori* eradication therapy using repeated serological tests has only been shown to be useful if a period of several months is maintained between tests [11-13].

Culture of *H. pylori* in biopsy specimens has very high specificity and allows testing for antibiotic susceptibility but has relatively low sensitivity and is labour-intensive [14]. Histological identification of *H. pylori* in biopsy specimens has long been considered to be the clinical standard for the diagnosis of *H. pylori* infection. A high density of *H. pylori* is readily apparent on routine hematoxylin and eosin (H&E) stains but detection of a lower density of bacteria may require additional staining techniques [15]. *H. pylori* is more easily visualised with immunohistochemical *H. pylori* antibody stains than with the standard H&E staining. However, the use of immunohistochemical (IHC) stains adds time and expense to the diagnostic evaluation for *H. pylori* and is therefore not routinely performed.

The interaction between *H. pylori* infection and the use of non-steroidal anti-inflammatory drugs (NSAIDs) in the development of gastroduodenal ulcers remains unclear. In a meta-analysis of 16 endoscopic studies in NSAID users from various countries, uncomplicated gastric ulcer disease was twice as common in *H. pylori* positive patients as in *H. pylori* negative patients [16]. However, the rate of *H. pylori* infection in patients with NSAID associated gastric ulcers is significantly lower than in those with non-NSAID associated gastric ulcers [17]. Furthermore, while eradication of *H. pylori* infection in NSAID-naïve patients prior to NSAID therapy reduces the risk of ulcer development, it does not do so in current NSAID users [18-20]. This was also confirmed in a recent randomized, double blind, placebo controlled clinical trial, in which we found that eradication of *H. pylori* infection did not reduce the incidence of endoscopic gastroduodenal ulcers in *H. pylori* seropositive patients currently taking NSAIDs for rheumatic diseases [21].

*H. pylori* infection has been shown to induce cyclooxygenase (COX)-2 expression in the gastric mucosa, which persists during active *H. pylori* infection [22-25]. It has been suggested that COX-2 plays an immunosuppressive role in *H. pylori* gastritis [26]. Conversely, in *H. pylori* infected mice, NSAID treatment has been shown to significantly decrease the degree of gastric inflammation [27]. It is therefore possible that in patients with *H. pylori* infection, concurrent NSAID treatment may affect levels of gastric inflammation and may consequently affect the serological response. While several studies have investigated the time course of *H. pylori* antibody titers after *H. pylori* eradication therapy, none have been conducted in NSAID users [9,11-13,28].

This study presents a post-hoc investigation into *H. pylori* IgG-antibody titer changes following *H. pylori* eradication therapy in NSAID users. In patients participating in the before mentioned *H. pylori* eradication in NSAID users trial, we measured *H. pylori* IgG-antibody titers and titer changes in order to diagnose successful *H. pylori* eradication [29]. We further compared *H. pylori* IgG-antibody titers, H&E stains, IHC stains and *H. pylori* culture results in follow-up biopsies from *H. pylori*-positive NSAID-users randomized to eradication treatment or placebo, to determine the sensitivity and specificity of these different methods in NSAID users. Furthermore, we determined whether adding IHC stains to H&E stains improves the histological identification of *H. pylori* in these patients.

## Methods

### Study design

The methods of the primary randomized, double blind, placebo controlled clinical trial have been previously described in more detail^21^. Between May 2000 and June 2002, patients between the ages of 40 and 80 years with a rheumatic disease requiring NSAID treatment, were recruited and included in the study if tested positive for *H. pylori* on serological testing. During the study, no change in NSAID-therapy was permitted, but there was no restraint on other medication. Exclusion criteria were previous *H. pylori* eradication therapy and severe concomitant disease.

After stratification by concurrent use of gastroprotective agents (proton pump inhibitors, H2 receptor antagonists or misoprostol, but not prokinetics, or antacids), patients were randomly assigned to receive either *H. pylori* eradication therapy with omeprazole 20 mg, amoxycillin 1000 mg, and clarithromycin 500 mg (OAC) twice daily for 7 days or placebo. Patients with an allergy for amoxycillin were randomized in a separate stratum to receive omeprazole 20 mg, metronidazol 500 mg and clarithromycin 250 mg (OMC) or placebo therapy twice daily for one week. Randomization to consecutive patient numbers was done in proportions of 1:1, in blocks of four from a computer-generated list. The study centers were provided with individually sealed packages containing the treatment for each patient. Each centre received entire blocks to be used sequentially. Rheumatologists were not practicing in more than one center. The study medication was given in a double blind, double dummy manner. Active and placebo preparations were identical in appearance. The employees of the VU University Medical Center pharmacy who packaged the medication only knew the assignment. It was disclosed to the treating physician only in case of emergency. All study personnel and participants were blinded to treatment assignment for the duration of the study.

After 3 months patients underwent gastroduodenal endoscopy, during which 4 antrum biopsies and 4 corpus biopsies were taken for culture and histological examination. After 3 and 12 months, blood samples were taken for repeated serological testing. Immunohistochemical staining was only available for a subset of patients recruited at the Medisch Spectrum Twente hospital in Enschede, the Netherlands. The study protocol was approved by the Institutional Ethical Review Board of all participating hospitals and all patients gave written informed consent.

### Serology

Serological testing for *H. pylori* IgG-antibodies was performed using a commercially available enzyme-linked immunosorbent assay (ELISA) kit (Pyloriset® new EIA-G, Orion Diagnostica, Espoo, Finland). Results were considered positive if the antibody titers were ≥250 International Units per mL (IU/mL), according to the manufacturer’s guidelines. This assay has been assessed, in a population similar to the population in the presented trial, and has proven a sensitivity and specificity in the Netherlands of 98-100% and 79-85%, even in patients on acid suppressive therapy [11,30,31].

### Culture

Biopsy specimens of corpus and antrum taken during endoscopy were inoculated onto Columbia agar (Becton Dickinson, Cockeysville, MD, USA) with 10% lysed horse blood (Bio Trading, Mijdrecht, The Netherlands), and onto Columbia agar with *H. pylori* selective supplement (Oxoid, Basingstoke, UK). Media were then incubated for 72 hours at 37°C under microaerophilic conditions (5% O_2_, 10% CO_2_ and 85% N_2_). The isolated colonies of *H. pylori* were identified by Gram stain showing spiral-shaped Gram-negative rods, producing urease rapidly, with positive catalase and oxidase tests.

### Histology

Biopsy specimens were stained for Hematoxylin and Eosin (H&E) according to the standard procedure. For immunohistochemical (IHC) staining, the slides were heated in an autoclave (Kavoklave, Prestige Medical Ltd, UK) in a citric-acid solution (pH = 6 to 121–126°C during 30 minutes for antigen retrieval. The slides were then incubated in a Shandon Sequenza Immunostaining Center (Thermo Electron Corporation, the Netherlands) with a polyclonal rabbit IgG anti-Helicobacter pylori antibody (DakoCytomation, Denmark, dilution 1:300), followed by biotinylated goat anti-polyvalent antibody (LabVision Corporation, USA), strepavidin peroxidase (LabVision Corporation, USA) and Liquid DAB + substrate chromogen system (DakoCytomation, Denmark), and counterstained with hematoxylin.

All stained biopsy specimens of corpus and antrum taken during endoscopy were examined by a single expert pathologist who was blinded for clinical data, treatment allocation and other test results.

### Gold standard definition

As the gold standard for *H. pylori* infection in this study, at 3 months a patient was defined as being *H. pylori* positive on the basis of a positive culture for *H. pylori* or, in the case of a negative culture, a positive examination of either H&E or IHC stains in combination with *H. pylori* IgG-antibody titers persistently ≥ 250 IU/mL. At 12 months, a patient was defined as being *H. pylori* positive on the basis of a positive culture for *H. pylori* or, in the case of a negative culture, a positive examination of either H&E or IHC stains in biopsy samples at 3 months in combination with *H. pylori* IgG-antibody titers persistently ≥ 250 IU/mL at 12 months.

### Statistical analysis

Continuous variables with a normal distribution were expressed as mean with standard deviation (SD), and continuous variables with a non-normal distribution as median with interquartile range (IQR). Differences between groups were analysed using Students t-test, Mann–Whitney U test, Pearson’s Chi-square test or Fisher’s Exact test in case of low expected values. For all analyses *P* < 0.05, two sided, was considered significant. All analyses were performed with SPSS for Windows, version 19.0 (SPSS, Chicago, IL, USA). Receiver Operating Characteristic (ROC) curves and likelihood ratios were analysed with MedCalc for windows, version 12.1.3.0. Differences in the proportions of patients were analyzed with 95% confidence interval using the Confidence Interval Analysis software for Windows (version 2.2.0).

## Results

A total of 347 patients were included in the present study. The treatment groups (172 patients in the eradication group and 175 patients receiving placebo) were similar in terms of demographics, rheumatic disease, NSAIDs and other drug use. Our eligibility criteria resulted in a study group with mainly inflammatory rheumatic diseases (rheumatoid arthritis 61%, spondyloarthropathy 8%, psoriatic arthritis 7%, osteoarthritis 9%, other 15%). The most commonly used NSAIDs were diclofenac (29%), naproxen (18%), and ibuprofen (13%). The mean age was 60 years (SD 10), 61% was female. Twenty-two patients had a known allergy for amoxicillin and received metronidazole instead (10 patients) or placebo (12 patients). Forty-eight percent used a gastroprotective drug (7% H2 receptor antagonists (H2RA), 37% proton pump inhibitors (PPI), 7% misoprostol, 3% used a combination of these).

At baseline, Anti-*H. pylori* IgG antibodies were present in all 347 patients (median titre 1689 (IQR 700–3732). At three months, data on both culture and histology were available in 305 patients; 152 in the eradication group and 153 in the placebo group. In two cases only culture data were available and in 1 case only histology was available. All three cases met the criteria for *H. pylori*-positivity and were found in the placebo group. A total of 32 patients (with no significant differences between eradication and placebo groups) refused the 3-month endoscopy, withdrew informed consent, or could not undergo endoscopy because of adverse events. Seven patients used anticoagulant therapy, ruling out biopsy sampling in accordance with the study protocol, and in one patient no biopsy specimens could be obtained because of discomfort requiring early completion of the procedure.

The results of *H. pylori* detection by each of the different tests are shown in Table 1. Out of the 152 patients who had been treated with *H. pylori* eradication therapy, 141 (93%) had a negative culture, and of the 153 patients who had been receiving placebo, 54 (35%) had a negative culture (*P* < 0.001). Out of the 152 patients who had been treated with *H. pylori* eradication therapy, 133 (88%) had a negative H&E stain, compared to 41 (27%) of the 153 patients who had been receiving placebo (*P* < 0.001). In the subgroup (with statistically similar baseline characteristics as the whole population, data not shown) of 68 patients in which IHC stains were performed, 29 (85%) of the 34 patients who had been treated with *H. pylori* eradication therapy had a negative IHC stain, compared to 7 (21%) of the 34 patients in the placebo group (*P* < 0.001). There were no differences between patients using gastroprotection compared to patients who did not take gastroprotective drugs for the presence of *H. pylori* by culture or histology (p = 0.454).

**Table 1:** **Results of *H. pylori *detection by each test**

***Test***		***Positives (%)***	***Negatives (%)***
***T=3months***			
Culture (N=305)	Eradication	11 (7)	141 (93)
	Placebo	99 (65)	54 (35)
H&E stains (N=305)	Eradication	19 (12)	133 (88)
	Placebo	112 (73)	41 (27)
IHC stains (N=68)	Eradication	29 (85)	5 (15)
	Placebo	7 (20)	27 (79)
Serology (N=203)	Eradication	92 (91)	9 (9)
	Placebo	94 (92)	8 (8)
***T=12months***			
Serology (N=304)	Eradication	96 (64)	55 (36)
	Placebo	138 (90)	15 (10)

H&E: hematoxylin and eosin, IHC: immunohistochemistry. Positive serology was defined as *H. pylori* IgG-antibody titers ≥ 250 IU/mL.

According to the gold standard criteria, a patient could be either *H. pylori* positive or *H. pylori* negative. The sensitivity, specificity, positive predictive values (PPV) and negative predictive values (NPV) of each test were calculated for the whole group and also differentiated for preceding *H. pylori* eradication therapy or placebo, as is shown in Table 2. For the combined analysis of H&E and IHC stains, results were positive if either test was positive or results were negative if both tests were negative. According to the gold standard criteria for *H. pylori* infection, *H. pylori* eradication was successful in 133 (89.9%) of the 148 patients who had been treated with *H. pylori* eradication therapy, while 120 (78.9%) of the 152 patients who had been receiving placebo remained *H. pylori* positive. Gold standard criteria could not be calculated in 4 patients in the eradication group en 1 in the placebo group because of missing or negative culture results, or missing serology data in combination with available histology results.

**Table 2:** **Results of the sensitivity, specificity, positive predictive value (PPV) and negative predictive value (NPV) of each test; for the total study group and differentiated for preceding *H. pylori *eradication therapy or placebo**

***Test***	***Sensitivity (%)***	***Specificity (%)***	***PPV (%)***	***NPV (%)***
Culture				
Total	82	100	100	87
Eradication	73	100	100	97
Placebo	83	100	100	62
H&E stains				
Total	93	99	99	94
Eradication	93	99	93	99
Placebo	92	100	100	78
***Subgroup of 68 patients***				
IHC stains				
Total	100	95	94	100
Eradication	100	94	60	100
Placebo	100	100	100	100
H&E+IHC				
Total	100	92	91	100
Eradication	100	90	50	100
Placebo	100	100	100	100

H&E: hematoxylin and eosin, IHC: immunohistochemistry.

### Serology

**Figure 1:**
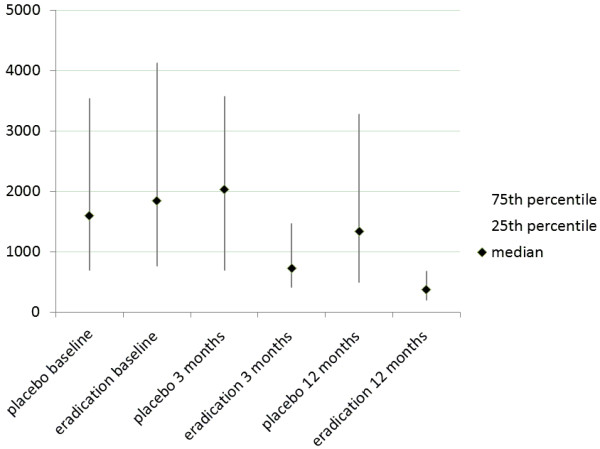
**Median (black diamond) and interquartile range (grey line) of *H. pylori *IgG-antibody titers in IU/mL for the eradication and placebo groups, at baseline, 3 months and 12 months after eradication therapy.**

At baseline, *H. pylori* IgG-antibody titers varied from 250 IU/mL to 19029 IU/mL with a median of 1689 IU/mL (interquartile range (IQR) 700 to 3732 IU/mL) with no significant differences in titers between the groups assigned to *H. pylori* eradication therapy or to placebo (P = 0.39). At endoscopy at 3 months, *H. pylori* IgG-antibody titers varied from 126 IU/mL to 12800 IU/mL, with a median of 1190 IU/mL (IQR 500 to 2820 IU/mL). Patients who had been treated with *H. pylori* eradication therapy had lower *H. pylori* IgG-antibody titers than those treated with placebo; eradication group (n = 101) median 730 IU/mL (IQR 415 to 1461 IU/mL) and placebo group (n = 102) median 2026 IU/mL (IQR 700 to 3571 IU/mL) (median difference −907, 95% CI −1356 to −460, P < 0.001 Figure 1). At serological testing at 12 months, patients who had been treated with *H. pylori* eradication therapy had lower *H. pylori* IgG-antibody titers than those treated with placebo; eradication group (n = 151) median 370 IU/mL (IQR 200 to 672 IU/mL) and placebo group (n = 153) median 1340 IU/mL (IQR 490 to 3272 IU/mL) (median difference −778, 95% CI −1128 to −466, P < 0.001 Figure 1).

At 3 months, *H. pylori* IgG-antibody titers had dropped below the 250 IU/mL threshold for positivity in 17/203 (8.4%) patients; 9/101 (9%) in the eradication group and 8/102 (8%) in the placebo group (*P* = 0.78). At 12 months, *H. pylori* IgG-antibody titers had dropped below the 250 IU/mL threshold for positivity in 70/304 (23%) patients; 55/151 (36%) in the eradication group and 15/153 (10%) in the placebo group (*P* < 0.05), Table 1.

The absolute change in *H. pylori* IgG-antibody titers from baseline to 3 months (titer at baseline minus titer at 3 months) did differ significantly between the groups; eradication group median change 980 IU/mL (IQR 190 to 2720 IU/mL) and placebo group median change −26 IU/mL (IQR −605 (elevation of titer) to 870 IU/mL) (median difference 1006, 95% CI 654 to 1471, *P < 0.001*). The change in *H. pylori* IgG-antibody titers from baseline to 12 months also differed significantly between the groups; eradication group median change 1010 IU/mL (IQR 363 to 2917 IU/mL) and placebo group median change 167 IU/mL (IQR −337 (elevation of titer) to 1625 IU/mL) (median difference 913, 95% CI 547 to 1362, *P* < 0.001).

Compared to baseline, at 3 months *H. pylori* IgG-antibody titers were median 55% lower (IQR 24% to 72%) in the eradication group and median 0.9% lower (IQR −32% to 40%) in the placebo group (median difference 46%, 95% CI 34% to 60%, *P* < 0.001). Compared to baseline, at 12 months *H. pylori* IgG-antibody titers were median 77 % lower (IQR 48% to 88%) in the eradication group and median 22 % lower (IQR −34% to 56%) in the placebo group (median difference 46%, 95% CI 36 to 58, *P* < 0.001).

**Figure 2:**
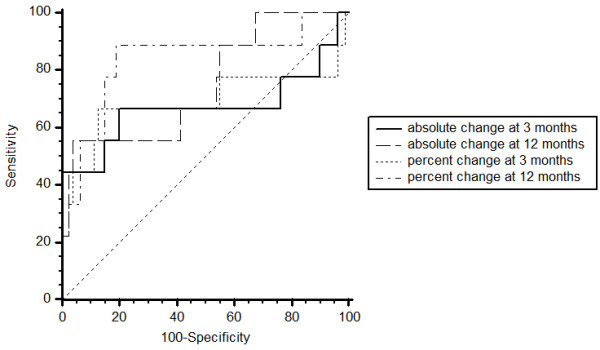
**Comparison of ROC curves for absolute and percent change of *H. pylori *-IgG antibody titers at 3 and 12 months after eradication therapy.**

Using the predefined *H. pylori* IgG-antibody titer cut-off point of ≥250 IU/mL, serological testing for *H. pylori* IgG-antibodies at endoscopy at 3 months was found to be highly sensitive (99%) but with very poor specificity (15%), especially following *H. pylori* eradication therapy (10%). Arguably, the absolute or percent change in *H. pylori* IgG-antibody titers from baseline represent better methods for evaluating success of *H. pylori* eradication. Figure 2 presents the Receiver Operating Characteristic (ROC) curves for absolute and percent change in *H. pylori* IgG-antibody titers after 3 and 12 months, associated with a negative result for the gold standard criteria for *H. pylori* infection. Percent change scores had better diagnostic power in identifying *H. pylori* negative patients at both 3 and 12 months, with area under the ROC curves (AUCs) of 0.62 (95% CI 0.52 to 0.72, *P =* 0.343) for absolute change and 0.70 (95% CI 0.59 to 0.79, *P* = 0.085) for percent change at 3 months and 0.73 (95% CI 0.65 to 0.80, *P* = 0.0016) for absolute change and 0.83 (95% CI 0.76 to 0.89, *P* < 0.0001) for percent change at 12 months. The optimal cut-off point at 3 months for percent change in *H. pylori* IgG-antibody titers was 21 %, corresponding to a sensitivity of 64% (95% CI 31% to 89%) and specificity of 81% (95% CI 71% to 89%), negative Likelihood ratio 0.45 (95% CI 0.2 to 1.1), positive Likelihood ratio 3.3 (95% CI 2.1 to 5.2). The optimal cut-off point at 12 months for percent change in *H. pylori* IgG-antibody titers was 58%, corresponding to a sensitivity of 87% (95% CI 60% to 98%) and specificity of 74% (95% CI 65% to 81%), negative Likelihood ratio 0.18 (95% CI 0.05 to 0.7), positive Likelihood ratio 3.3 (95% CI 2.6 to 4.1).

## Discussion

Following *H. pylori* eradication therapy or placebo, histological examination of gastric mucosal tissue biopsies provided good sensitivity and specificity ratios for evaluating success of *H. pylori* eradication therapy. In the subgroup with both IHC and H&E staining, IHC was slightly superior to H&E. Following eradication therapy both staining methods provided 100% sensitivity and also very high specificity. A combined analysis of H&E and IHC stains, in which results were positive if either test was positive or results were negative if both tests were negative, did not improve sensitivity while the number of false positive test results increased. Culture of *H. pylori* in gastric biopsy specimens has very high specificity but relatively low sensitivity [5,32]. In the present study, culture provided 100% specificity and 82% sensitivity. However, after *H. pylori* eradication therapy sensitivity dropped to 73% due to an increasing percentage of false negative cultures. Culture of *H. pylori* therefore does not appear to be very useful for evaluating success of *H. pylori* eradication therapy. In clinical practice, invasive tests for confirmation of eradication should only be used in cases where repeat endoscopy is indicated, for example in patients with gastric ulcer. In all other cases non-invasive test should be employed for follow-up after *H. pylori* eradication treatment [33].

The choice of a gold standard affects test results of all other tests. According to the guidelines for clinical trials in *H. pylori* infection, a reliable gold standard should consist of at least 2 methods based on different principles for detecting *H. pylori* infection [5,34]. In the present study, a patient was also considered *H. pylori* positive if culture alone was positive, in view of its absolute specificity. The gold standard in the present study corresponds to acceptable criteria.

Other accurate and relatively inexpensive non-invasive tests that may also be considered for the evaluation of success of *H. pylori* eradication therapy are serology, 13C-urea breath tests and stool antigen tests [33]. While the 13C-urea breath test may have better accuracy (>90%), the serology test used in this study was less expensive and readily available in all study centres [35]. At the time of the study, stool antigen tests were not yet widely available in the Netherlands. PPI usage (in this study 48 % of the population) may result in false negative test results in both invasive and non-invasive tests, such as culture, histology and 13C-urea breath testing, and should therefore be stopped two weeks before testing [36]. This does not apply for serological testing. Besides, stopping PPI in a population of chronic NSAID users would be non-ethical in a trial setting.

This study shows that in NSAID users, percent change in *H. pylori* IgG-antibody titers has better diagnostic power in identifying *H. pylori* negative patients at both 3 and 12 months than absolute change in *H. pylori* IgG-antibody titers. Repeated serological testing using a cut-off point of 21% decrease in *H. pylori* IgG-antibody titers after 3 months and 58% after 12 months has sufficiently high sensitivity and specificity to be useful for evaluating the success of *H. pylori* eradication therapy. Other groups have found high sensitivity and specificity ratios for percent decrease in *H. pylori* IgG-antibody titers using cut-off points of 25% at 6 months and 40% at 3 to 6 months [10,11,37]. Using a predefined *H. pylori* IgG-antibody titer cut-off point of 250 IU/mL, repeated serological testing for *H. pylori* IgG-antibodies was found to have little diagnostic value.

Overall, NSAID use did not seem to influence *H. pylori* eradication rates or serological testing for *H. pylori* IgG-antibodies, when compared to other studies with patients who do not take NSAIDs [5,32]. Although studies on *H. pylori* IgG serology are not new, there is some data available in which has been shown that NSAID treatment significantly decreases the degree of gastric inflammation [22-25]. However in some studies aspirin and NSAID possibly suppresses the growth of *H. pylori* and may influence diagnostic testing and increase its susceptibility to the antibiotics [38-40]. It is therefore possible that in patients with *H. pylori* infection, concurrent NSAID treatment may affect levels of gastric inflammation and may consequently affect the serological response. While several studies have investigated the time course of *H. pylori* antibody titers after *H. pylori* eradication therapy, none have been conducted in NSAID users yet. Theoretically, if NSAID treatment decreases the degree of gastric inflammation and subsequently affects the serological response, one would not expect to find many false positive test results. However, such an effect still cannot be ruled out because in the present study, a relatively strong decline in *H. pylori* IgG-antibodies was noted 3 months after *H. pylori* eradication (median 55% decline at 3 months and median 77% decline at 12 months), compared to other studies. A previous longitudinal analysis of *H. pylori* IgG-antibody titers following successful *H. pylori* eradication demonstrated a mean decline of 26% at 3 months, 43% at 6 months, and 55% at nine months follow-up, after which titers appeared to plateau at approximately 50% compared to baseline [28].

## Conclusions

In the present study in NSAID taking patients, following *H. pylori* eradication therapy or placebo, histological examination of gastric mucosal tissue biopsies provided good sensitivity and specificity ratios. The H&E and IHC staining methods provided comparable high sensitivity and specificity but combining IHC and H&E did not improve results. A percentual *H. pylori* IgG-antibody titer change has better sensitivity and specificity than an absolute titer change or a predefined *H. pylori* IgG-antibody titer cut-off point for evaluating success of *H. pylori* eradication therapy.

## Competing interests

The author(s) declare that they have no competing interests.

## Authors' contributions

HEV carried out analyses and drafted the manuscript. HDL participated in the design of the study and coordination, carried out the analyses and drafted the manuscript. MVL conceived of the study, and participated in its design and coordination and helped to draft the manuscript. JVB carried out pathological assessments. KSS participated in the design of the study and helped to draft the manuscript. WFL conceived of the study, and participated in its design and coordination and helped to draft the manuscript. JWB conceived of the study, and participated in its design and coordination and helped to draft the manuscript. EJK conceived of the study, and participated in its design and coordination and helped to draft the manuscript. HMH participated in the design of the study and helped to draft the manuscript. MJ participated in the design of the study and helped to draft the manuscript. BAD conceived of the study, and participated in its design and coordination and helped to draft the manuscript. All authors read and approved the final manuscript.

## Pre-publication history

The pre-publication history for this paper can be accessed here:

http://www.biomedcentral.com/1471-230X/12/133/prepub
